# Tanshinone IIA Facilitates Efficient Cartilage Regeneration under Inflammatory Factors Caused Stress via Upregulating LncRNA NEAT1_2

**DOI:** 10.3390/biomedicines11123291

**Published:** 2023-12-12

**Authors:** Jingjing Sun, Wei Chen, Zheng Zhou, Xin Chen, You Zuo, Jiaqian He, Hairong Liu

**Affiliations:** 1 College of Biology, Hunan University, Changsha 410082, China; jingjingsun@hnu.edu.cn (J.S.); you-zuo@hotmail.com (Y.Z.); hejiaqian@hnu.edu.cn (J.H.); 2 College of Material Science and Engineering, Hunan University, Changsha 410082, China; chenwei690@hnu.edu.cn (W.C.); chenxin_hnu@hnu.edu.cn (X.C.)

**Keywords:** osteoarthritis, tanshinone IIA, NEAT1_2, cartilage defects repair, inflammatory

## Abstract

(1) Background: Osteoarthritis (OA) is a crippling condition characterized by chondrocyte dedifferentiation, cartilage degradation, and subsequent cartilage defects. Unfortunately, there is a lack of effective medicines to facilitate the repair of cartilage defects in OA patients. In this study, we investigated the role of lncRNA NEAT1_2 in maintaining the chondrocyte phenotype and identified tanshinone IIA(TAN) as a natural medicine that enhances NEAT1_2 levels, resulting in efficient cartilage regeneration under inflammatory cytokines. (2) Methods: The transcriptional levels of NEAT1_2 and cartilage phenotype-related genes were identified by RT-qPCR. The siRNA interference approach was utilized to silence NEAT1_2; the Alamar Blue assay was performed to determine chondrocyte viability under inflammatory conditions. To evaluate the concentrations of collagen type II and glycosaminoglycans distributed by chondrocytes in vitro and in vivo, immunohistochemical staining and Safranin O staining were used. (3) Results: IL-1β suppresses NEAT1_2 and genes related to the chondrocytic phenotype, whereas TAN effectively upregulates them in a NEAT1_2-dependent manner. Consistently, TAN alleviated chondrocyte oxidative stress inhibited cartilage degradation by modulating the relevant genes and promoted efficient cartilage regeneration in vitro and in vivo when chondrocytes are exposed to inflammatory cytokines. (4) Conclusions: TAN enhances the expression of NEAT1_2 inhibited by IL-1β and affects the transcription of chondrocytic phenotype-related genes, which promotes cartilage regeneration in an inflammatory environment.

## 1. Introduction

Osteoarthritis (OA) is a prevalent degenerative joint disease that threatens millions of patients, and it involves various histological lesions around the infrapatellar fat pad and synovium, periarticular muscles, ligaments, subchondral bone, and especially articular cartilage [1,2]. Compared with other tissues, articular cartilage mainly consists of chondrocytes and the large amount of extracellular matrix (ECM) which they produce. Chondrocytes are responsible for maintaining the balance between ECM anabolism and catabolism. However, numerous factors such as inflammatory cytokines, abnormal mechanical stimuli, chondrocyte apoptosis, and oxidative stress disrupt chondrocyte physiology and the balance of matrix transitions, which in turn leads to matrix loss and tissue degeneration, resulting in OA [3,4]. The inflammatory cytokines, especially interleukin (IL)-1β and tumor necrosis factor-α (TNF-α), play a dominant role in the process of chondrocyte dedifferentiation. They downregulate chondrocytic phenotype-related genes such as SRY-Box Transcription Factor 9 (*SOX9*) and Aggrecan (*ACAN*) genes as well as collagen type II (*COLII*), contributing to the dedifferentiation of chondrocytes and subsequent cartilage defects [5]. Currently authorized therapies are still primarily concerned with symptomatic alleviation. Consequently, there is an urgent requirement to further comprehend the role of inflammation in the development and progression of OA, as well as to develop medical treatments to repair OA cartilage defects.

Tanshinone IIA (TAN) is a lipophilic diterpene extracted from *Salvia miltiorrhiz* Bunge, a herbal medicine with many active ingredients, which has anti-inflammatory [6], antioxidant [7], and vascular endothelial cell-protective properties [8]. It has been suggested that TAN is effective in the treatment of chronic inflammatory diseases [9,10,11,12]. For instance, the treatment of OA in SD rats with TAN resulted in a significant decrease in Mankin’s score (*p* < 0.002), a significant decrease in the levels of inflammatory factors such as IL-1β, TNF-α and inducible nitric oxide synthase (*iNOS*) in the serum of rats, and effective relief of OA symptoms [9]. TAN restricts the proliferation, migration, and invasion of rheumatoid arthritis (RA) fibroblast-like synoviocytes and effectively inhibits the increases in some matrix metalloproteinases and pro-inflammatory factors induced by TNF-α, thereby suppressing the inflammatory response and preventing knee joint destruction [10]. TAN resists the damage to chondrocytes by inflammatory stimuli and maintains their activities [11]. Moreover, TAN promotes the proliferation of chondrocytes and enhances the regeneration of cartilage tissue in vitro and in vivo [12]. It seems like that TAN may influence the cellular response to inflammatory-caused stress via regulating the expression of related proteins and ncRNAs.

Publications suggest that long noncoding RNAs (lncRNAs) play essential roles in OA, and bioactive small molecules, like TAN, may alter the expression of lncRNAs, including MALAT1, PVT1, HOTAIR, H19, and NEAT1 [13,14]. Nuclear-enriched abundant transcript 1 (NEAT1) plays an essential role in the advancement of several diseases, particularly inflammation [15,16,17]. Recently, lncRNA NEAT1 has been implicated in the regulatory processes of OA [18,19]. NEAT1_2 is one of the two heterodimeric transcripts of NEAT1, which serves a structural biological function as the core backbone of paraspeckles that participate in various cellular stress responses [20]. Hence, it is worth testing whether the bioactivity of TAN links with the expression of NEAT1_2 to maintain the cartilage phenotype.

In this study, we investigated how TAN influences the phenotype of chondrocytes in an inflammatory environment and prevents chondrocytes from oxidative stress and apoptosis induced by inflammatory stimuli. To explore the potential therapeutic implications, we also examined whether TAN affects cartilage regeneration through histological staining both in vivo and in vitro.

## 2. Materials and Methods

### 2.1. Cell Separation and Culture

The animal experiments involved in this study were approved by the Experimental Animal Ethics Committee, The Second Xiangya Hospital, Central South University, China. Rabbit chondrocytes were isolated from the articular cartilage of legs, which were obtained from New Zealand white rabbits (1-week-old, Hunan Slake Kingda Laboratory Animal Co., Changsha, China). Briefly, rabbit cartilage tissues were washed three times with PBS containing 1% antibiotics and digested with trypsin (Sangon Biotech (Shanghai) Co., Shanghai, China) at 37 °C for 30 min to remove other tissues and cells. Then, cartilage was cut into 1 mm^3^ pieces, and these pieces were digested with 0.2% collagenase II (Gibco, Carlsbad, CA, USA) at 37 °C for 8 h. Afterward, the digested cartilage pieces were filtered through a strainer and transferred to cell culture dishes for further incubation.

The human-derived chondrocytes were a gift from Dr Fang Bairong of the Second Xiangya Hospital of Central South University. Chondrocytes were extracted as described above. The chondrocytes and chondrosarcoma cells (SW1353) (Institute of Biochemistry and Cell Biology, Shanghai, China) were cultured in DMEM high glucose culture medium containing 10% fetal bovine serum (Gibco, Carlsbad, CA, USA) and 1% dual antibodies (penicillin/streptomycin sulfate) at 37 °C. Cells were all treated in the exponential growth phase.

### 2.2. Preparation of Silk Fibroin Scaffolds and TAN-Loaded Silk Fibroin Scaffolds

Silk fibroin scaffolds and TAN-loaded silk fibroin scaffolds were prepared according to the methods in our published publications and the experimental steps were optimized [12]. Specifically, adding 0.02 M Na_2_CO_3_ solution over the cocoon boiling for 30 min, adding distilled water at 75 °C to wash twice, then drying the silk at 60 °C to a constant weight. LiBr solutions were added to dissolve the silk. The mixture should be dialysis and then centrifugation. Eventually, the collected silk protein supernatant was stored at 4 °C.

TAN (purity 98%, Aladdin, Shanghai, China) was dissolved in anhydrous ethanol and combined with the prepared silk fibroin solution to provide final TAN concentrations of 0, 5, 10, 20, and 40 µg/mL. The mixture was added into each well of a 96-well cell culture plate to reach the depth of 2 mm, and then frozen at −20 °C for 6 h, then transferred to −2 °C for 48 h. Following freezing, samples were lyophilized for 24 h to obtain TAN-loaded silk fibroin scaffolds and silk fibroin scaffolds. These scaffolds were named SF, SF/T5, SF/T10, SF/T20, and SF/T40, respectively, according to the concentration of TAN before mixing.

### 2.3. Characterization and Biocompatibility of TAN-Loaded Silk Fibroin Scaffolds

#### 2.3.1. SEM

Scanning electron microscopy (SEM) was used to observe the microscopic morphology of tested samples (FEI Quanta 200, FEI Company, Hillsboro, OR, USA). The pore size distribution of the samples is also counted based on the microcosmograms.

#### 2.3.2. Drug Release Properties of TAN-Loaded Silk Fibroin Scaffolds

To determine the TAN release capacity of the TAN-loaded silk fibroin scaffolds, the samples were incubated in PBS solution at pH 7.4 for 35 days and the supernatants were collected. The cumulative release of TAN was measured by UV spectrophotometry (UV2600, Shimadzu, Japan).

#### 2.3.3. Cell Proliferation Assays

Rabbit chondrocytes (1 × 10^5^) were inoculated on every tested sample. The proliferation of chondrocytes was evaluated by Alamar Blue assay [21], which was cultured in culture medium with different influence factors for 1, 3, 5, and 7 days. The reduction rate measured on the first day was normalized to characterize the proliferation ploidy of chondrocytes in this study.

### 2.4. Isolation and Extraction of Total RNA

Chondrocytes and SW1353 cells were incubated with normal culture medium and total RNA was extracted as the control samples. SW1353 cells and chondrocytes were cultured with culture medium, which contained 10 ng/mL IL-1β, for 24 h, then the proper volume of TAN solution was added into this IL-1β-containing culture medium (to obtain a final concentration of 2 µg/mL) to incubate these cells for 6 h, 12 h, 24 h, and 48 h.

We divided the TAN-loaded silk fibroin scaffolds into normal and inflammation groups according to the culture medium. The samples in the normal group were incubated with normal medium for 10 days. Samples of the inflammatory group were cultured in normal medium for 3 days and then cultured in medium containing 10 ng/mL IL-1β and 10 ng/mL TNF-α for 7 days.

We lysed the cells using Trizol (Invitrogen, Carlsbad, CA, USA) to extract total RNA and reverse transcription of total RNA with a reverse transcription kit (K1683, Thermo Scientific, Waltham, MA, USA). Following that, the samples were subjected to reverse transcription-quantitative polymerase chain reaction (RT-qPCR) and used the gene of glyceraldehyde-3-phosphate dehydrogenase (GAPDH) as the control. The relevant primer sequences are listed in Table 1 and Table 2.

### 2.5. Small Interfering RNA (siRNA) Transfection

To investigate how lncRNA NEAT1_2 influences the chondrogenic phenotype under the stress of inflammatory factors, SW1353 cells were transfected with a small interfering RNA targeting NEAT1_2 or with a control siRNA to silence NEAT1_2. Lipofectamine 2000 reagent (Invitrogen, Carlsbad, CA, USA) was employed to transfect SW1353 cells. After 24 h of IL-1β stimulation, we added culture medium containing TAN and IL-1β for 24 h and collected treated SW1353 cells. Total RNA was extracted from collected cells for further analysis.

### 2.6. Cartilage Regeneration Evaluation In Vitro and In Vivo

To evaluate the efficiency of cartilage regeneration, we ribbit inoculated chondrocytes (2 × 10^6^) on the scaffolds and then cultured them for 2 or 4 weeks. Following incubation, samples were sliced and treated with hematoxylin and eosin (H&E) (Solarbio, Beijing, China) and safranin-O (SO) (Solarbio, Beijing, China) staining. The glycosaminoglycan (GAG) contained in samples was quantified using the colorimetric method of dimethyl methylene blue (DMMB).

To test the efficiency of cartilage regeneration in vivo, ribbit chondrocytes were seeded on the scaffolds and were cultured in vitro for 3 days. All tested samples were incubated with medium containing IL-1β and TNF-α for one week. A total of 9 nude mice (6 weeks old, males, Hunan Slake Kingda Laboratory Animal Co., Changsha, China) were assigned randomly, and then the sample was implanted subcutaneously into each mouse. The implanted samples were collected 4 weeks after surgery. After a gross morphological examination was carried out, histological analysis (H&E, SO, and immunohistochemistry of type II collagen) of each sample was performed to assess the efficiency of cartilage regeneration in vivo.

### 2.7. Statistical Analysis

All data in this study are expressed as the standard deviation (mean ± SD.) and are obtained from at least three independent samples or experiments (*n* ≥ 3). Differences between groups were statistically assessed using one-way analysis of variance (ANOVA) or Student’s *t*-test. Statistical analyses were performed by GraphPad Prism version 8.0 (GraphPad Software, San Diego, CA, USA). The significant differences were judged at the *p* < 0.05 (* *p* < 0.05, ** *p* < 0.01, *** *p* < 0.001).

## 3. Results

### 3.1. Under IL-1β Caused Stress TAN Upregulates the Expression of NEAT1_2

**Figure 1:**
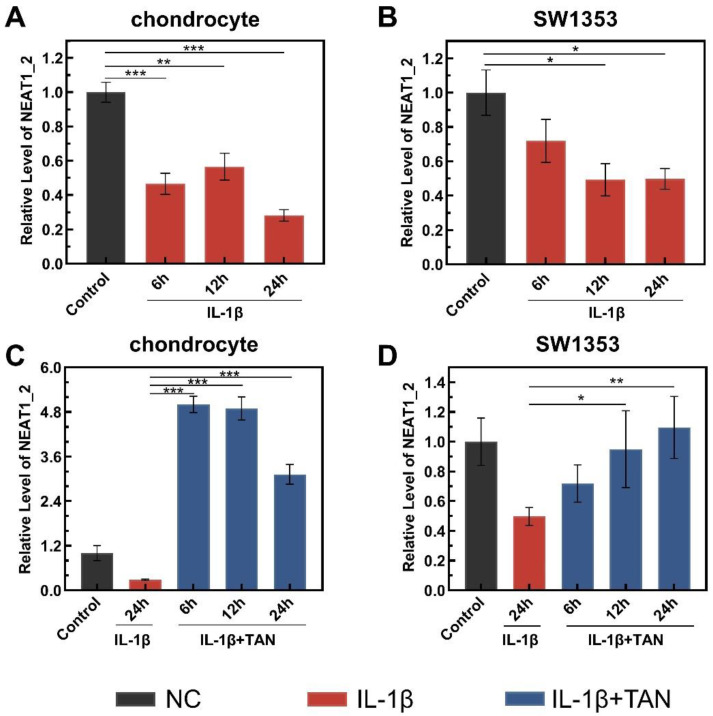
TAN upregulates NEAT1_2 transcription in response to IL-1β stimulation. The relative RNA level of NEAT1_2 in chondrocytes (**A**) and SW1353 (**B**) under IL-1β stimulation, and the relative RNA level of NEAT1_2 in chondrocytes (**C**) and SW1353 (**D**) after IL-1β combined with TAN treatment. Values were normalized, and the RNA expression levels in untreated cells were set to 1. The data were obtained from 3 independent experiments (*n* = 3), and the error bars indicate SD. * *p* < 0.05, ** *p* < 0.01, *** *p* < 0.001, by two-tailed Student’s *t*-test. NC: normal culture medium; IL-1β: culture medium containing IL-1β; IL-1β + TAN: culture medium containing IL-1β and tanshinone IIA.

It was suggested that as the backbone of paraspeckles NEAT1_2 is involved in the cellular replication stress response, possibly including the cellular response to inflammatory factors [22,23]. We speculated that the dedifferentiation of chondrocytes in OA patients may relate to the alteration of NEAT1_2. By using human chondrocytes, with incubation with IL-1β, the level of NEAT1_2 was significantly reduced (Figure 1A). Since human chondrocytes can be cultured for limited generation, the level of NEAT1_2 significantly declined under IL-1β-caused stress in a chondrosarcoma cell line (Figure 1B), SW1353, which will be used as the model to carry out further investigation. Based on publications, it seems likely that bioactive small molecules, which are purified from plants or herbs that are used to treat OA, may influence the expression of NEAT1_2 [24,25]. Interestingly, it has been revealed that TAN significantly upregulates NEAT1_2 in chondrocytes in response to IL-1β-caused stress (Figure 1C,D).

### 3.2. TAN Enhances the Transcription of Chondrocyte Phenotype Genes by Upregulating NEAT1_2 Expression under IL-1β Caused Stress

**Figure 2:**
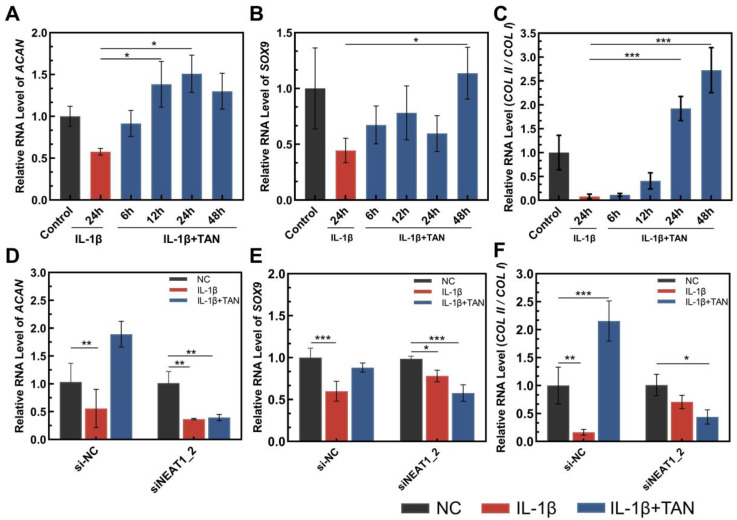
The relative RNA level of genes listed and the ratio of *COL II*/*COL I*. (**A**) The relative RNA level of *ACAN* in the cases indicated; (**B**) the relative RNA level of *SOX9* in the cases indicated; and (**C**) the ratio of *COL II*/*COL I* in the cases indicated. (**D**) The *ACAN* level following IL-1β alone and IL-1β combined with tanshinone IIA treatment in cells indicated; (**E**) the *SOX9* level following IL-1β alone and IL-1β combined with tanshinone IIA treatment in cells indicated; (**F**) the ratio of *COL II*/*COL I* following IL-1β alone and IL-1β combined with tanshinone IIA treatment in cells indicated. Values were normalized, and the RNA expression levels in untreated cells (control) were set to 1. The data were obtained from 3 independent experiments (*n* = 3). * *p* < 0.05, ** *p* < 0.01, *** *p* < 0.001, one-way analysis of variance. NC: normal culture medium; IL-1β + TNF-α: culture medium containing IL-1β and TNF-α; IL-1β + TNF-α + TAN: culture medium containing IL-1β, TNF-α, and tanshinone IIA.

Given that NEAT1_2 is known to be involved in cellular responses to different types of stress, it is plausible to consider that TAN may potentially upregulate NEAT1_2 under IL-1β-induced stress, leading to an increase in the transcription of genes associated with the chondrocytic phenotype. In the case of IL-1β-caused stress, the mRNA level of *SOX9*, *ACAN*, and the *COL II*/*COL I* ratio in cells were significantly increased following incubation with TAN (Figure 2A–C). To investigate whether IL-1β caused stress in the upregulation of *SOX9*, *ACAN*, and the *COL II*/*COLI* ratio directly related to the TAN-induced upregulation of lncRNA NEAT1_2, an additional siRNA was used to knockdown NEAT1_2 in the same condition (Figure S1). It was displayed that with the knockdown of NEAT1_2 expression, TAN lost the capacity to upregulate *SOX9*, *ACAN*, and the *COL II*/*COL I* ratio under IL-1β-caused stress (Figure 2D–F).

### 3.3. Preparation and Characterization of TAN-Loaded SF Scaffolds for Further Investigation

**Figure 3:**
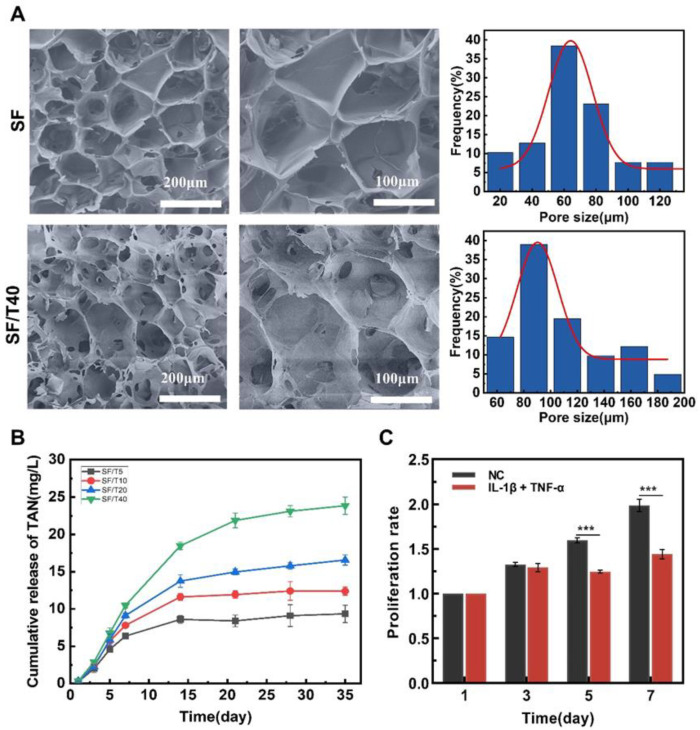
Preparation and characterization of SF scaffolds and TAN-loaded SF scaffolds. (**A**) Scanning electron microscope micrograph of SF and SF/T40. (**B**) Cumulative release of TAN from TAN-loaded silk fibroin scaffold in PBS (pH = 7.4) at 37 °C. (**C**) Proliferation rates of chondrocytes grown on silk fibroin scaffolds incubated with normal culture medium and IL-1β along with TNF-α containing culture medium, respectively (*n* = 4). The data were obtained from at least 3 independent experiments (*n* = 3). *** *p* < 0.001, by two-tailed Student’s *t*-test.

To achieve continuous exposure of chondrocytes to TAN and considering that the two-dimensional culture on cell culture plates is not suitable for long-term maintenance of the chondrocyte phenotype, which hinders the evaluation of cartilage regeneration, we established a TAN-loaded SF scaffold model to further our research. The SF and SF/T40 scaffolds revealed an internally interconnected three-dimensional porous structure in SEM images (Figure 3A). There was no significant difference in the pore size distributions of SF and SF/T40, which were all in the range of 60–120 μm (Figure 3A). It is demonstrated that the addition of TAN had no effect on the pore size of the scaffolds, and the range of the pore size was appropriate for chondrocyte migration and nutrient transport. After 35 days of cumulative drug release testing, all samples sustained TAN release; specifically, TAN release was rapid during the pre-culture period, reaching an inflection point after 7 days and a plateau TAN concentration in PBS solution after 28 days (Figure 3B). In addition, the cumulative release of TAN over time is also presented in Table S1. It is suggested that TAN-loaded SF scaffolds revealed the ability to gently release TAN while continually protecting the chondrocyte phenotype for cartilage repair during inflammatory factors incubation.

To verify the effect of inflammatory factors on chondrocyte proliferation, the culture medium containing IL-1β and TNF-α was used to treat chondrocytes seeded on SF scaffolds for 7 days. The data displayed that IL-1β combined with TNF-α significantly inhibited the proliferation of chondrocytes (Figure 3C), implying that the combination of IL-1β and TNF-α is suitable for representing the inflammatory factors of OA patients influencing chondrocytes.

### 3.4. TAN Upregulates the Transcription of Genes Facilitating Cartilage Regeneration under Inflammatory Factors Induced Stress

**Figure 4:**
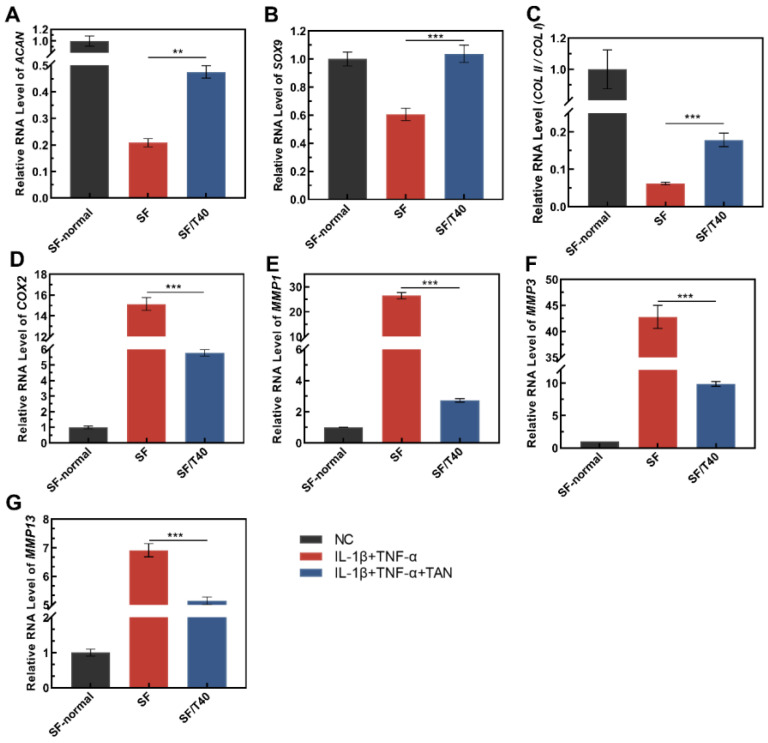
TAN influences the transcription of genes related to chondrogenesis and cartilage degradation in response to inflammatory stimuli. (**A**) *ACAN*, (**B**) *SOX9*, (**C**) the ratio of *COLII*/*COLI*, (**D**) *COX2*, (**E**) *MMP1*, (**F**) *MMP3*, (**G**) *MMP13*. The data were obtained from at least 3 independent experiments (*n* = 3), ** *p* < 0.01, *** *p* < 0.001, by two-tailed Student’s *t*-test. NC: normal culture medium; IL-1β + TNF-α: culture medium containing IL-1β and TNF-α; IL-1β + TNF-α + TAN: culture medium containing IL-1β, TNF-α, and tanshinone IIA.

Whether TAN releasing from TAN-loaded SF scaffolds similarly affects adhered chondrocytes was validated by using rabbit chondrocytes, since SW1353 is a cancer cell, which is not suitable for testing cartilage regeneration. Following incubation with the culture medium containing IL-1β and TNF-α for 7 days, *SOX9*, *ACAN*, and the *COLII*/*COL I* ratio were significantly downregulated, which is consistent with results obtained from cell culture plates (Figure 4A–C). Compared with chondrocytes seeded on SF scaffolds, in chondrocytes adhered on TAN-loaded SF scaffolds, the transcript levels of *SOX9*, *ACAN*, and the *COLII*/*COL I* ratio were significantly upregulated by TAN following incubation with the culture medium containing IL-1β and TNF-α. Within these TAN-loaded SF scaffolds, which were SF/T5, SF/T10, SF/T20, and SF/T40, the SF/T40 scaffold displayed the highest influence on genes transcriptional regulation in chondrocytes with the induction of IL-1β and TNF-α (Figure 4A–C). Hence, the SF/T40 scaffold was selected for further investigation.

The maintenance of the chondrocyte phenotype by TAN may also be achieved by influencing the transcription of other cartilage-related genes, and the transcription of genes related to cartilage extracellular matrix degradation was detected with the same treatment applied to chondrocytes. Under IL-1β and TNF-α caused stress, the transcription of *COX-2* was significantly upregulated, and the enhanced expression of *COX-2* subsequently promotes the secretion of matrix metalloproteinases (*MMPs*), which degrade the extracellular matrix of cartilage tissue (Figure 4D). With the induction of IL-1β and TNF-α, the transcript levels of *MMP1*, *MMP3*, and *MMP13* in chondrocytes were significantly upregulated, and TAN significantly suppressed the transcript levels of *MMP1*, *MMP3*, and *MMP13* in cells adhered on SF/T40 scaffolds compared with those on SF scaffolds (Figure 4E–G).

### 3.5. TAN Attenuates the Aggravation of IL-1β and TNF-α Induced Stress and Inhibits Apoptosis in Chondrocytes

**Figure 5:**
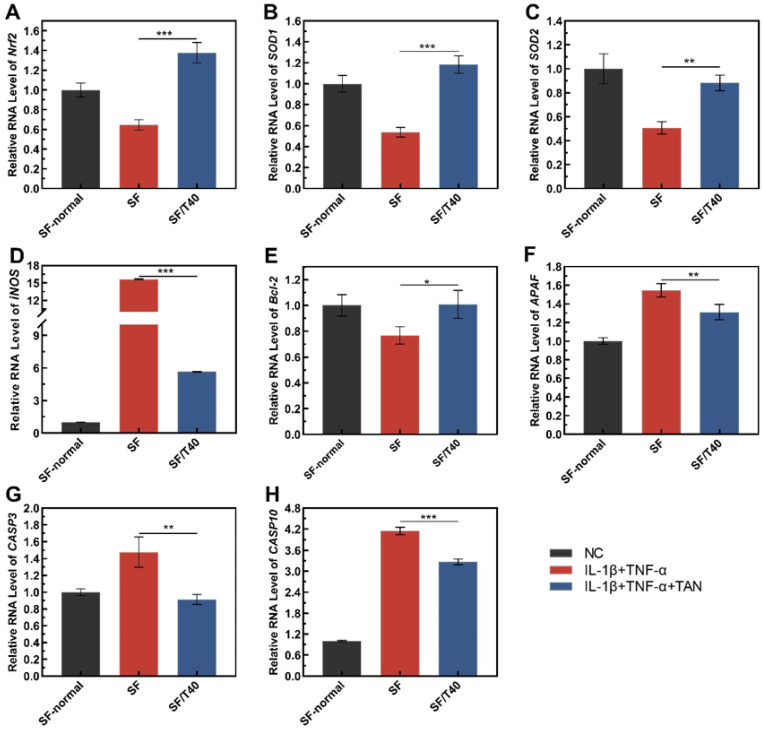
TAN affects the transcription of oxidative stress- and apoptosis-related genes in response to inflammatory stimuli. (**A**) *Nrf2*, (**B**) *SOD1*, (**C**) *SOD2*, (**D**) *iNOS*, (**E**) *Bcl-2*, (**F**) *APAF*, (**G**) *CASP3*, and (**H**) *CASP10*. The data were obtained from at least 3 independent experiments (*n* = 3). * *p* < 0.05, ** *p* < 0.01, *** *p* < 0.001, by two-tailed Student’s *t*-test. NC: normal culture medium; IL-1β + TNF-α: culture medium containing IL-1β and TNF-α; IL-1β + TNF-α + TAN: culture medium containing IL-1β, TNF-α, and tanshinone IIA.

Following chondrocyte exposure to IL-1β and TNF-α, the transcription of *Nrf2*, *SOD1*, and *SOD2* were substantially repressed in chondrocytes and *iNOS* was significantly upregulated (Figure 5A). Similarly, the IL-1β and TNF-α-caused suppression of *Nrf2*, *SOD1*, and *SOD2* was significantly reversed by TAN, which reduces the oxidative stress for cells. The significant upregulation of *iNOS* induced by IL-1β and TNF-α was declined by TAN, implying that it blocked the aggravation of stress in cells.

Meanwhile, TNF-α may cause apoptosis of chondrocytes, and whether TAN works in suppressing chondrocyte apoptosis in response to the stimuli of IL-1β and TNF-α was tested with the same method. (Figure 5D–F). In contrast to chondrocytes seeded on the SF scaffold, the transcription of *Bcl-2* was upregulated by TAN in cells adhered to SF/T40 scaffolds. The transcription of *CASP3*, *CASP10*, and *APAF* was significantly upregulated in chondrocytes following IL-1β and TNF-α induction, but in the same conditions, upregulation of *CASP3*, *CASP10*, and *APAF* was significantly reduced by TAN, which may inhibit the initiation and progress of apoptosis.

### 3.6. TAN Promotes Cartilage Regeneration In Vitro following the Induction of IL-1β and TNF-α

Based on all results described above, TAN maintains the chondrocytic phenotype of chondrocytes and keeps chondrocytes alive and active under IL-1β and TNF-α caused stress, suggesting that TAN promotes cartilage regeneration even in a circumstance with the presence of inflammatory factors. To confirm this hypothesis, samples with seeded chondrocytes were incubated with a culture medium containing IL-1β and TNF-α for 1 week, and then, two or four weeks of incubation with a normal culture medium was applied. Samples, which were SF-normal scaffolds with seeded chondrocytes, were incubated with a normal culture medium for 5 weeks and were regarded as the positive control.

**Figure 6:**
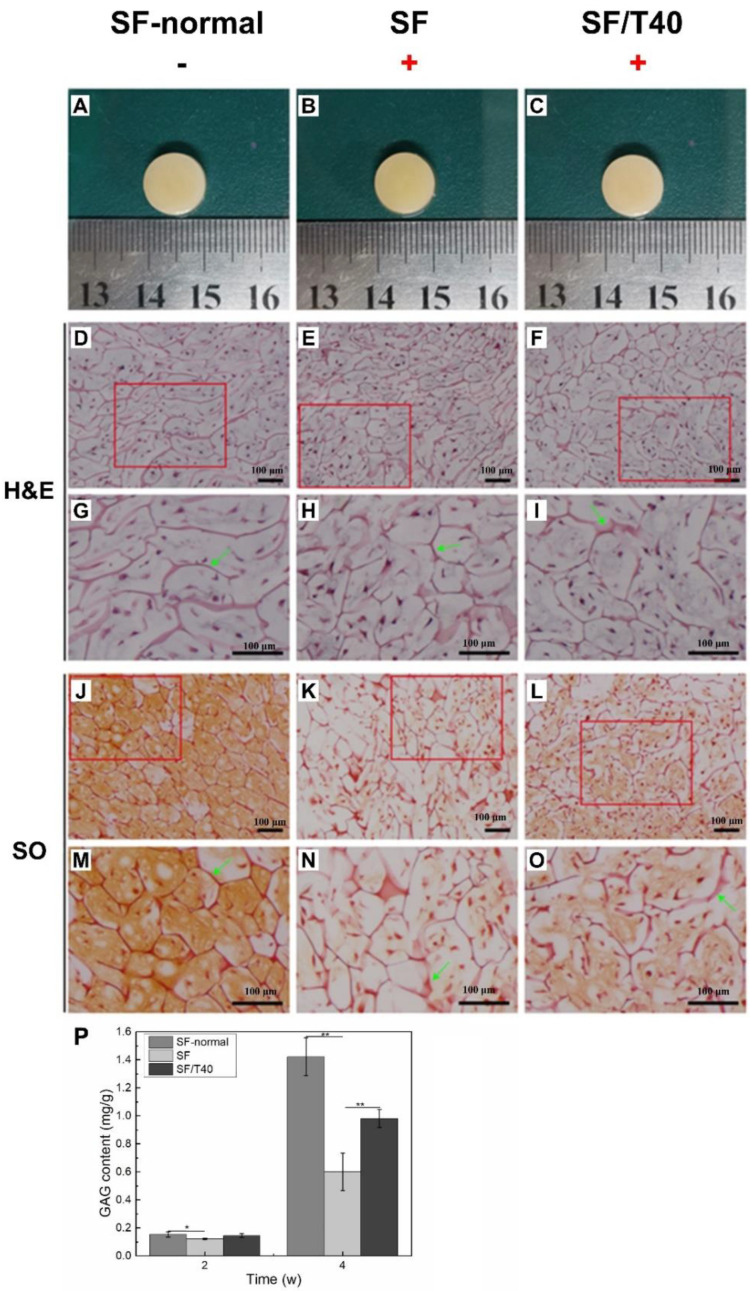
TAN promotes cartilage regeneration in vitro. Gross morphology (**A**–**C**), H&E staining (**D** –**I**), SO staining (**J**–**O**) and GAG content (**P**) of samples (*n* = 3) after 4 weeks of in vitro culture following IL-1β and TNF-α stimuli for 1 week. (‘+’ indicates addition of IL-1β + TNF-α to the culture environment. Green arrows indicate scaffolds. The red box is the corresponding zoom area). The data were obtained from at least 3 independent experiments (*n* = 3). * *p* < 0.05, ** *p* < 0.01, by two-tailed Student’s *t*-test.

After 5 weeks of incubation with a normal culture medium, many living chondrocytes and specific glycosaminoglycan (GAG) deposition were observed, indicating that cartilage tissue was regenerated (Figure 6A,D,G,J,M). With IL-1β and TNF-α induction for 1 week, it seems that barely any cartilage tissue was generated resulting from extremally low GAG deposition (Figure 6B,E,H,K,N).

With the assistance of TAN, the GAG deposition nearly covered the area of samples, demonstrating that chondrocytes maintained their phenotype and were able to generate cartilage tissue following the induction of IL-1β and TNF-α for 1 week (Figure 6C,F,I,L,O). Furthermore, the deposition of GAG in every sample was quantified, by which the efficiency of cartilage tissue regeneration can be roughly estimated. Without disturbing with IL-1β and TNF-α for 1 week, the GAG content was 1.42 ± 0.14 mg/g in positive control samples after 4 weeks, which was significantly higher than other samples that experienced IL-1β and TNF-α induction (Figure 6P). The GAG content of samples was dropped to 0.60 ± 0.13 mg/g after these samples were treated with IL-1β and TNF-α induction for 1 week, but with the assistance of TAN, the GAG content in samples significantly increased to 0.98 ± 0.06 mg/g with the same treatment. Although the GAG deposition in the two-week samples is not as high as that in the four-week samples, it demonstrates the same trend (Figure S2).

### 3.7. TAN Enhances Cartilage Regeneration In Vivo following IL-1β and TNF-α Induction

**Figure 7:**
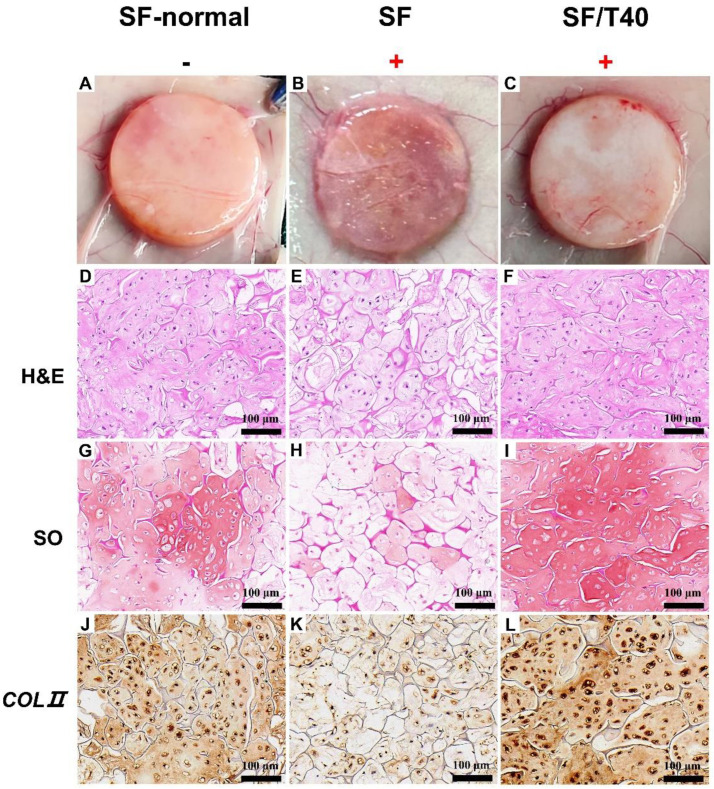
TAN enhances cartilage regeneration in vivo. Gross morphology (**A**–**C**), H&E staining (**D**–**F**), SO staining (**G**–**I**), and collagen II immunohistochemistry staining (**J**–**L**) of samples after 4 weeks of subcutaneous implantation in nude mice (*n* = 3) following IL-1β and TNF-α induction for 1 week. (‘+’ indicates addition of IL-1β + TNF-α to the culture environment).

To verify whether TAN regulates the transcription of genes to facilitate cartilage regeneration under IL-1β and TNF-α-caused stress in vivo, tested scaffolds seeded with 2 × 10^6^ chondrocytes were implanted into nude mice for 4 weeks following IL-1β and TNF-α induction for 1 week. During the feeding period, the nude mice showed normal vital activity and no swelling or inflammation around the samples. Samples were collected after 4 weeks of subcutaneous implantation, and all samples retained their original shape (Figure 7A–C). Without IL-1β and TNF-α treatment, chondrocytes were distributed evenly in the samples (Figure 7D), and efficient cartilage regeneration was observed everywhere in the samples (Figure 7G,J). Following IL-1β and TNF-α treatment, living chondrocytes slightly reduced (Figure 7E), but the efficiency of cartilage regeneration dramatically declined, which is inferred from the reduced deposition of GAG and Col II (Figure 7H,K) compared with samples without IL-1β and TNF-α treatment (Figure 7G,J). However, with TAN functioning living chondrocytes evenly distributed in samples (Figure 7F), the high intensity of SO staining and collagen II immunohistochemistry staining reflected that efficient cartilage regeneration happened in samples (Figure 7I,L) despite IL-1β and TNF-α treatment. In conclusion, with the assistance of TAN, efficient cartilage regeneration can be achieved under the stress caused by inflammatory factors, suggesting that TAN can be applied to clinically treat OA patients to repair cartilage defects in the future.

## 4. Discussion

Increased degradation of cartilage extracellular matrix leading to cartilage defects is a typical symptom in OA patients, and the key to effective treatment of degenerative OA is the progressive restoration of damaged articular cartilage [26,27]. There are currently no approved medicines that have been demonstrated to be effective in repairing defective tissue and delaying the progression of OA. There is accumulating proof that the pathogenesis of OA has been associated with the generation of pro-inflammatory mediators, cartilage matrix breakdown, chondrocyte oxidative stress, and apoptosis [28,29]. In this study, we report that TAN can upregulate the transcription of chondrocytic phenotype-related genes under inflammatory stress, and this upregulation is likely achieved through lncRNA NEAT1_2. Moreover, we found that TAN also upregulates other cartilage-related genes to alleviate chondrocyte oxidative stress and apoptosis and promote cartilage regeneration both in vitro and in vivo.

According to recent studies, long non-coding RNAs may play a crucial role in the prevention of the development of OA [5,14,30]. In particular, NEAT1 interacts with microRNAs to influence chondrocyte proliferation, migration, and apoptosis along with extracellular matrix (ECM) secretion, which in turn affects OA [25,31]. We revealed that NEAT1_2 plays a key role in chondrocytes responding to the stress caused by inflammatory factors and dedifferentiation. Under the stress caused by IL-1β, the level of NEAT1_2 is significantly reduced, as well as the downregulation of chondrocyte phenotype-related genes like *SOX9* and *ACAN* and the subsequent dedifferentiation of chondrocytes. The *SOX9* and *ACAN* genes and the collagen type II *(COLII)*/collagen type I *(COLI)* ratio as well as the matrix metalloproteinase gene (MMPs) are mainly associated with the chondrocyte phenotype, and their alteration implies an imbalance between ECM synthesis and degradation [32]. Interestingly, TAN reversed the transcription of genes related to the chondrocyte phenotype under inflammatory stress. Plant-derived active compounds have emerged in recent years as attractive pharmacological candidates for targeting lncRNAs and attaining disease treatments by altering lncRNA up- or down-regulation, and TAN is no exception [13,33,34]. Therefore, we knocked down NEAT1_2 by siRNA interference and verified that TAN’s effect on cartilage phenotypes can be exerted via modifying NEAT1_2.

It has been well documented that IL-1β and TNF-α induce oxidative stress in chondrocytes, causing chondrocyte dedifferentiation, apoptosis, and reduced proliferation, which fundamentally inhibits cartilage regeneration [35]. *Nrf2* is a major regulator of oxidative stress in chondrocytes, and its low expression links with the evolution and deterioration of OA [36]. In normal chondrocytes, *iNOS* exhibits a very low expression level, but its expression is significantly elevated in arthritic chondrocytes, catalyzing NO synthesis, and generating oxidative stress in cells, leading to further aggravation of joint inflammation [37]. Although the anti-inflammatory and antioxidant properties of TAN have been demonstrated to be effective in the treatment of neurological diseases as well as chronic diseases such as cardiovascular and nephritic diseases, only a handful of studies on the therapy of OA cartilage repair have been reported [38,39,40]. Our findings suggest that TAN relieved the oxidative stress by promoting the transcription of *Nrf2*, *SOD1*, and *SOD2* and repressing the transcription of *iNOS*, and initiation and TAN-blocked progress caused the upregulation of *Bcl-2* and downregulation of *CASP3*, *CASP10*, and *APAF*. Altogether, TAN may facilitate cartilage regeneration by maintaining the chondrocytic phenotype of chondrocytes and keeping chondrocytes alive and active under TNF-α and IL-1β-caused stress. Furthermore, the results of tissue stain in vitro and in vivo demonstrated that TAN stimulated the secretion of *COLII* and GAGs by chondrocytes in response to IL-1β and TNF-α-induced stress, successfully rescuing the loss of ECM degradation caused by inflammatory stimuli (Figure 6 and Figure 7).

To sum up, TAN alleviated the imbalance between ECM synthesis and degradation caused by IL-1β and TNF-α and inhibited the development of apoptosis and oxidative stress in cartilage chondrocytes, implying that TAN could maintain chondrocyte viability and promote cartilage regeneration in OA patients, and thus could be a promising drug for the treatment of OA in the future.

## 5. Conclusions

In summary, TAN upregulated the expression of lncRNA NEAT1_2 under IL-1β-caused stress, by which the downregulation of chondrocytic phenotype-related genes caused by IL-1β induction is significantly reversed and consequently maintains the chondrocytic phenotype of chondrocytes. Similarly, TAN inhibits the initiation and progress of apoptosis of chondrocytes and relieves the oxidative stress under IL-1β and TNF-α-caused stress by regulating the transcription of related genes. Following IL-1β and TNF-α induction for 1 week, TAN facilitates efficient cartilage regeneration in vitro and in vivo, suggesting that it can be an innovative strategy for treating OA patients in the future.

## Figures and Tables

**Table 1:** Primer sequence for RT-qPCR in humans.

Gene	5′-3′	Primer
*GAPDH*	forward	TTCGACAGTCAGCCGCATCTTCTT
	reverse	GCCCAATACGACCAAATCCGTTGA
*NEAT1_2*	forward	GGCCAGAGCTTTGTTGCTTC
	reverse	GGTGCGGGCACTTACTTACT
*COLⅡ*	forward	TCACGTACACTGCCCTGAAG
	reverse	TGACCCTCAAACTCATGCCTC
*COLⅠ*	forward	GGCAACAGCAGGTTCACTTAC
	reverse	AGTTAGAACCCCCTCCATCCC
*ACAN*	forward	TCGTGGTGAAAGGTGAGAGC
	reverse	CGTGGAGGAGCTGGTTTGAA
*SOX9*	forward	ACTCGCCCCAACAGATCGCC
	reverse	GCTGGAGTTCTGGTGGTCGGTG

**Table 2:** Primer sequence for RT-qPCR in rabbits.

Gene	5′-3′	Primer
*GAPDH*	forward	TTGTCGCCATCAATGATCCAT
	reverse	GATGACCAGCTTCCCGTTCTC
*SOX9*	forward	GCGTCAACGGCTCCAGCAAGA
	reverse	GCGTTGTGCAGGTGCGGGTAC
*COLII*	forward	GAGAGCCTGGGACCCCTGGAA
	reverse	CGCCTCCAGCCTTCTCGTCAA
*COLI*	forward	CTAGCCACCTGCCAGTCTTTA
	reverse	GGACCATCATCACCATCTCTG
*ACAN*	forward	GCTGCTACGGAGACAAGGATG
	reverse	CGTTGCGTAAAAGACCTCACC
*MMP1*	forward	TTCCAAAGCAGAGAGGCAATG
	reverse	CACCTGGGTTGCTTCATCATC
*MMP3*	forward	GTGATACGCAAGCCCAGGTGT
	reverse	CTCTTGGCAGATCCGGTGTGT
*MMP13*	forward	GTCTTCTGGCTCACGCTTTTC
	reverse	GGCAGCAACGAGAAACAAGTT
*iNOS*	forward	GCTGGAGCTGAAGTGGTACGC
	reverse	CTCCGATCTCTGTGCCCATGT
*APAF*	forward	TCGTGGTCTGCTGATGGTGCT
	reverse	TGCTGTTACGGCCTGTTTGGA
*SOD2*	forward	CAGAAGCACAGCCTCCCCGAC
	reverse	CCGTGGCGTTCAGGTTGTTCA
*COX2*	forward	CCATTGACCAGAGCAGGCAGA
	reverse	CTCGGCAGCCATCTCCTTCTC
*Bcl-2*	forward	CGGAAGGGACTGGACCAGAGA
	reverse	GCTGTCATGGGGATCACCTCC
*CASP3*	forward	AAGCCACGGTGATGAAGGAGT
	reverse	TCGGCAAGCCTGAATAATGAA
*Nrf 2*	forward	ATTCTTTCGGCAGCATCCTCT
	reverse	CTGGGTTCAGCTATGAAGGCA
*SOD1*	forward	GCACGGATTCCATGTCCACCA
	reverse	TCACATTACCCAGGTCGCCCA

Abbreviation: *GAPDH*: glyceraldehyde-3-phosphate dehydrogenase; *COL II*: Collagen type II; *COL I*: Collagen type I; *ACAN*: Aggrecan; *SOX9*: SRY-Box Transcription Factor 9; *MMP1*: Matrix metallopeptidase 1; *MMP3*: Matrix metallopeptidase 3; *MMP13*: Matrix metallopeptidase 13; *iNOS*: Inducible nitric oxide synthase; *APAF*: Apoptotic peptidase activating factor; *SOD2*: Superoxide dismutase 2; *COX2*: Cyclooxygenase-2; *Bcl-2*: B-cell lymphoma-2; *CASP3*: Caspase 3; *Nrf 2*: Nuclear factor erythroid 2-related factor 2; *SOD1*: Superoxide dismutase 1.

## Data Availability

Source data are available from the corresponding author upon reasonable request.

## Supplementary Materials

The following supporting information can be downloaded at: https://www.mdpi.com/article/10.3390/biomedicines11123291/s1, Figure S1: The efficiency of siRNA interfering with NEAT1_2 in SW1353 indicated. *** *p* < 0.001, by two-tailed Student’s *t*-test. Figure S2: TAN promotes cartilage regeneration in vitro. Gross morphology (A–C), H&E staining (D–I) and SO staining (J–O) of samples (*n* = 3) after 2 weeks of in vitro culture following IL-1β and TNF-α stimuli for 1 week. (‘+’ indicates addition of IL-1β + TNF-α to the culture environment. Green arrows indicate scaffolds. The red box is the corresponding zoom area.) (‘+’ indicates addition of IL-1β + TNF-α to the culture environment. Green arrows indicate scaffolds. The red box is the corresponding zoom area.). Table S1: Cumulative release of TAN over time, in mg/L.
