# Supplementary material for: Tanshinone IIA Facilitates Efficient Cartilage Regeneration under Inflammatory Factors Caused Stress via Upregulating LncRNA NEAT1_2
Source: Biomedicines. 2023 Dec 12;11(12):3291. doi: 10.3390/biomedicines11123291 (PMC10741062; doi:10.3390/biomedicines11123291)
Supplement: Supplementary file 1 [file biomedicines-11-03291-s001.zip › biomedicines-2703520-supplementary.pdf]

Tanshinone IIA Facilitates Efficient Cartilage Regeneration under Inflammatory Factors Caused Stress via Upregulating LncRNA NEAT1_2

Jingjing Sun [1,†], Wei Chen [2,†], Zheng Zhou [1,*], Xin Chen [2], You Zuo [1], Jiaqian He [1] and Hairong Liu [2,*]

[1] College of Biology, Hunan University, Changsha 410082, China; jingjingsun@hnu.edu.cn (J.S.); you-zuo@hotmail.com (Y.Z.); hejiaqian@hnu.edu.cn (J.H.)
[2] College of Material Science and Engineering, Hunan University, Changsha 410082, China; chenwei690@hnu.edu.cn (W.C.); chenxin_hnu@hnu.edu.cn (X.C.)
* Correspondence: zhouzheng@hnu.edu.cn (Z.Z.); liuhairong@hnu.edu.cn (H.L.)
† These authors contributed equally to this work.

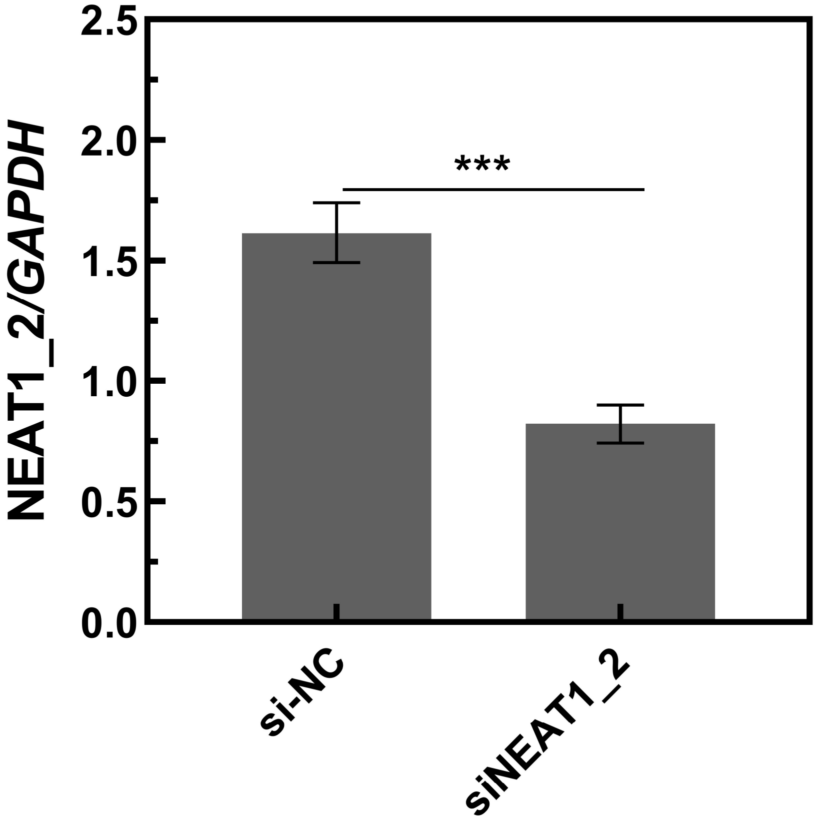

Figure S1. The efficiency of siRNA interfering with NEAT1_2 in SW1353 indicated.

**P* < 0.05, ***P* < 0.01, ****P* < 0.001, by two-tailed Student's *t* test.

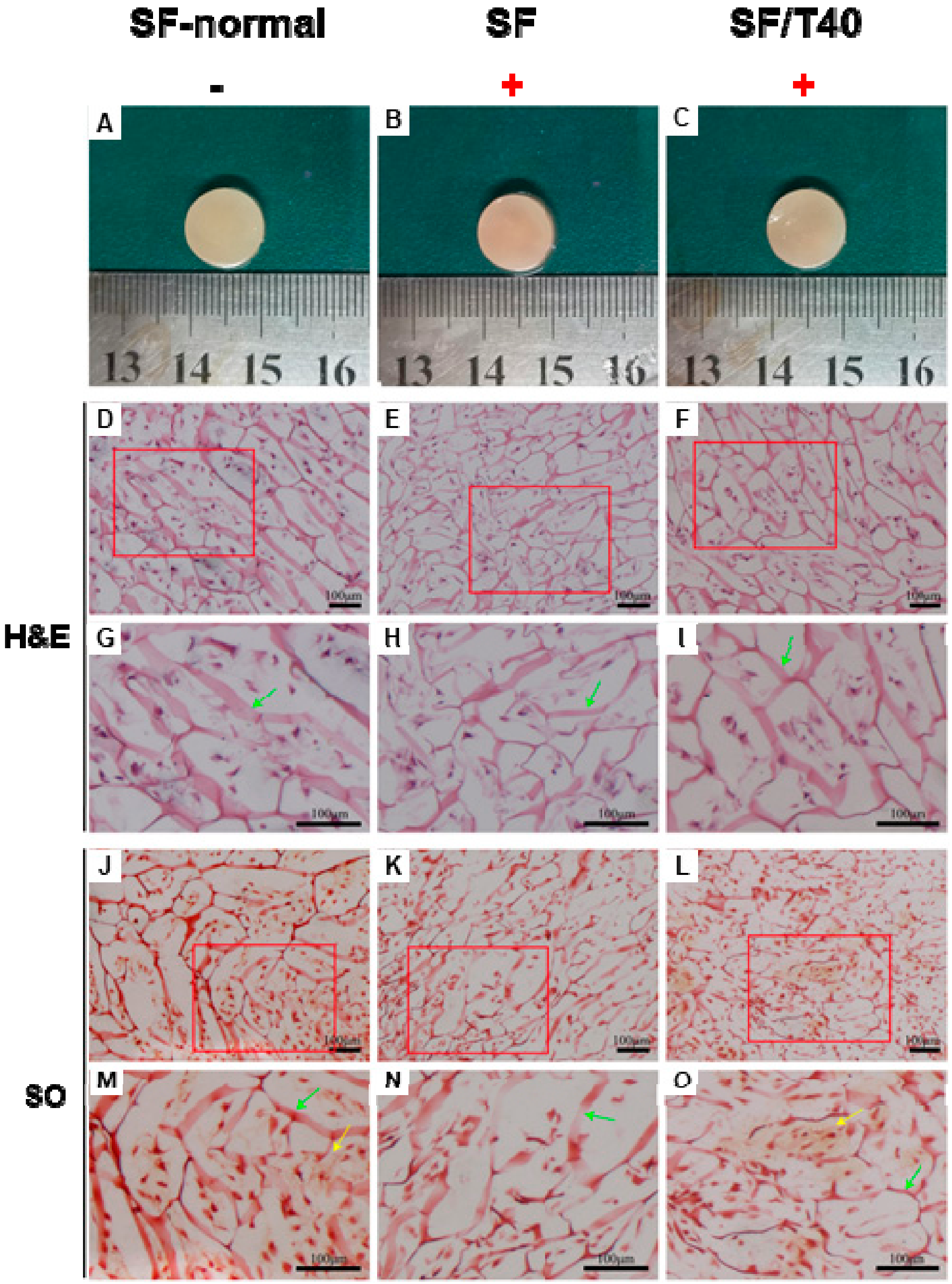

Figure S2. TAN promotes cartilage regeneration in vitro. Gross morphology (A-C), H&E staining(D-I) and SO staining (J-O) of samples(n=3) after 2 weeks of in vitro culture following IL-1β and TNF-α stimuli for 1 week. ('+' indicates addition of IL-1β + TNF-α to the culture environment. Green arrows indicate scaffolds. The red box is the corresponding zoom area.) ('+' indicates addition of IL-1β + TNF-α to the culture environment. Green arrows indicate scaffolds. The red box is the corresponding zoom area.).

Day \ Samples	SF/T5	SF/T10	SF/T20	SF/T40
1	0.24946	0.32334	0.32877	0.32551
3	1.99131	2.4259	2.25554	2.89526
5	4.57975	5.68796	5.8618	6.75272
7	6.35443	7.80052	9.11584	10.47347
14	8.602	11.61147	13.74489	18.47849
21	8.40417	11.92438	14.96654	21.87658
28	9.09952	12.41113	15.79226	23.11517
35	9.34289	12.38288	16.57453	23.85398

Table S1. Cumulative release of TAN over time, in mg/L.
